# 3D Team Care Management Trial for Cognitively Vulnerable Older Adults: Who Participates and How Does the Team Work?

**DOI:** 10.1093/geroni/igaa057.2677

**Published:** 2020-12-16

**Authors:** Richard Fortinsky, Caroline Stephens

## Abstract

Community-dwelling older adults often experience cognitive symptoms, and three common conditions that contribute to changes in cognition are dementia, depression and delirium. Despite the clinical inter-connectedness among these medical conditions, hereafter referred to collectively as cognitive vulnerability, little is known about the potential for success of clinical interventions that simultaneously address these conditions. From the perspective of older adults with cognitive vulnerability and their families, hospital admissions and emergency department (ED) visits are disorienting and often lead to declines in functional capacity and well-being, and significant family distress, threatening continued independent living. In this Symposium, we present details about an ongoing clinical trial testing a novel in-home, multidisciplinary team care management intervention for older adults with cognitive vulnerability and their families. This care management intervention led by nurse practitioners, called the3D Team care model, aims to help reduce ED visits and hospitalizations and achieve other health-related outcomes. The first presentation will provide study background and design features as well as characteristics of study participants. The next two presentations by the3D Team nurse practitioners will provide details about how the multidisciplinary team works, and how each team member provides interventions intended to address risk factors for adverse health outcomes. The fourth presentation by the3D Team community health educator will explain how needs related to social determinants of health are addressed. The Discussant will place this clinical trial within the broader context of multidisciplinary team care for older adults with cognitive vulnerability led by nurse practitioners trained in geropsychiatry.

